# ISG56/IFIT1 is primarily responsible for interferon-induced changes to patterns of parainfluenza virus type 5 transcription and protein synthesis

**DOI:** 10.1099/vir.0.046797-0

**Published:** 2013-01

**Authors:** J. Andrejeva, H. Norsted, M. Habjan, V. Thiel, S. Goodbourn, R. E. Randall

**Affiliations:** 1 School of Biology, Centre for Biomolecular Sciences, BMS Building, North Haugh, University of St Andrews, St Andrews, Fife, KY16 9ST, UK; 2 Kantonal Hospital St Gallen, Institute of Immunobiology, CH-9007 St Gallen, Switzerland; 3 Division of Basic Medical Sciences, St George’s, University of London, London SW17 0RE, UK

## Abstract

Interferon (IFN) induces an antiviral state in cells that results in alterations of the patterns and levels of parainfluenza virus type 5 (PIV5) transcripts and proteins. This study reports that IFN-stimulated gene 56/IFN-induced protein with tetratricopeptide repeats 1 (ISG56/IFIT1) is primarily responsible for these effects of IFN. It was shown that treating cells with IFN after infection resulted in an increase in virus transcription but an overall decrease in virus protein synthesis. As there was no obvious decrease in the overall levels of cellular protein synthesis in infected cells treated with IFN, these results suggested that ISG56/IFIT1 selectively inhibits the translation of viral mRNAs. This conclusion was supported by *in vitro* translation studies. Previous work has shown that ISG56/IFIT1 can restrict the replication of viruses lacking a 2′-*O*-methyltransferase activity, an enzyme that methylates the 2′-hydroxyl group of ribose sugars in the 5′-cap structures of mRNA. However, the data in the current study strongly suggested that PIV5 mRNAs are methylated at the 2′-hydroxyl group and thus that ISG56/IFIT1 selectively inhibits the translation of PIV5 mRNA by some as yet unrecognized mechanism. It was also shown that ISG56/IFIT1 is primarily responsible for the IFN-induced inhibition of PIV5.

## Introduction

Parainfluenza virus type 5 [PIV5; previously known as simian virus 5 (SV5)] is a member of the genus *Rubulavirus* in the subfamily *Paramyxovirinae* of the family *Paramyxoviridae* (Lamb & Parks, 2006). Like other paramyxoviruses, PIV5 is an enveloped virus with a non-segmented negative-sense RNA genome of 15 246 bases with seven tandemly linked genes that encode eight proteins. The RNA genome encodes, from the 3′ end, the nucleo- (NP), phospho- (P), V, matrix (M), fusion (F), small hydrophobic (SH), haemagglutinin–neuraminidase (HN) and large (L) proteins. All viral RNA synthesis is initiated at or close to the 3′ end of the genomic or antigenomic RNA. During transcription, the viral polymerase recognizes gene-start and gene-stop sequences in the genomic RNA, resulting in the generation of individual mRNAs that are also capped and polyadenylated by the viral polymerase. Failure of the polymerase to reinitiate at downstream gene-start sites, or disengagement of the polymerase from the genome, results in a transcriptional gradient, with the NP gene being transcribed the most frequently and the L gene the least. The second gene, the V/P gene, encodes both the P and the V proteins using an RNA editing mechanism. During replication, the polymerase must ignore the gene-start and gene-stop signals to make full-length copies of either the genomic or antigenomic template. As with other negative-strand RNA viruses, the polymerase does not replicate or transcribe naked RNA but rather uses the ribonucleoprotein complex as its template (for a general review of the molecular biology of paramyxovirsues, see Lamb & Parks, 2006, and for an extensive recent review on PIV5, see Parks *et al.*, 2011).

Like other viruses, to survive in nature, PIV5 has to circumvent, at least partially, the interferon (IFN) response (Randall & Goodbourn, 2008). The IFN response is triggered when virus infection results in the generation of molecules, such as dsRNA, that display molecular signatures, termed pathogen-associated molecular patterns (PAMPs), that are not found in uninfected cells and that are recognized by cellular receptors, termed pattern recognition receptors (PRRs) (Kumar *et al.*, 2011). The two most important cytoplasmic PRRs for detecting PAMPs produced by RNA viruses are retinoic acid-inducible gene I (RIG-I) and melanoma differentiation-associated gene 5 (MDA-5). RIG-I preferentially recognizes uncapped 5′-triphosphate RNA molecules that have a short stretch of dsRNA, whilst MDA-5 recognizes longer molecules of dsRNA that do not need to be 5′ triphosphorylated (Schmidt *et al.*, 2011). Upon binding their appropriate ligands, both RIG-I and MDA-5 initiate a signalling cascade that results in the secretion of IFN-β from the infected cells. The secreted IFN then acts in an autocrine and paracrine manner to upregulate the expression of hundreds of cellular genes, many of which have direct or indirect antiviral activity (Randall & Goodbourn, 2008).

PIV5 has a number of strategies to minimize the effectiveness of the IFN response. It encodes an IFN antagonist, the V protein, which limits the production of IFN and blocks IFN signalling (reviewed by Goodbourn & Randall, 2009; Ramachandran & Horvath, 2009). The V protein limits IFN induction by interacting directly with MDA-5, preventing it from binding dsRNA and thus preventing activation of the IFN induction cascade (Andrejeva *et al.*, 2004; Childs *et al.*, 2009). Furthermore, whilst the V protein does not bind directly to RIG-I, it indirectly inhibits RIG-I by binding to Laboratory of Genetics and Physiology 2 protein (LGP2, a helicase related to RIG-I and MDA-5), stabilizing the interaction of LGP2 with RIG-I, thereby inhibiting RIG-I (Childs *et al.*, 2012). In addition, it can act as a competitive inhibitor of TANK-binding kinase 1 (TBK1), reducing its ability to phosphorylate IFN regulatory factor 3 (IRF-3) (Lu *et al.*, 2008). The V protein also blocks IFN signalling by targeting signal transducer and activator of transcription 1 (STAT1) for proteasome-mediated degradation (Didcock *et al.*, 1999) by a mechanism that requires it to act as a bridge between a cellular E3 ubiquitin ligase and STAT1/STAT2 heterodimers (Parisien *et al.*, 2002; Precious *et al.*, 2005). In addition, to these mechanisms, PIV5 tightly controls the transcription and replication of its genome, thereby limiting the production of PAMPs that may activate the IFN induction cascade (Goodbourn & Randall, 2009; Parks *et al.*, 2011). Indeed, recent evidence suggests that PIV5 does this so successfully that PAMPs that activate the IFN induction cascade are not produced during normal virus replication (Killip *et al.*, 2011, 2012). Instead, PAMPs that activate the IFN response are produced primarily by defective interfering particles that are generated during aberrant virus replication (Chen *et al.*, 2010; Killip *et al.*, 2011).

Despite these IFN-evasive mechanisms and strategies, the ability of PIV5 to circumvent the IFN response is not absolute, as witnessed by the observation that plaques of PIV5 are significantly larger on cells that cannot produce and/or respond to IFN compared with IFN-competent cells (Carlos *et al.*, 2009; Young *et al.*, 2003). During plaque development, a few, rather than all, infected cells produce the IFN that induces an antiviral state in the surrounding uninfected cells (Chen *et al.*, 2010). Whilst the replication of PIV5 is severely restricted in cells in an IFN-induced antiviral state (Carlos *et al.*, 2005), nevertheless the virus manages to target STAT1 for proteasome-mediated degradation (Didcock *et al.*, 1999; Precious *et al.*, 2007). As a consequence, in the absence of continuous stimulation by IFN, the cell cannot maintain its antiviral state indefinitely, and within 24–48 h, the virus manages to establish a normal pattern of virus replication (Carlos *et al.*, 2009).

To study the effects of IFN on the replication of PIV5, we have developed systems in which cells that cannot produce IFN but can respond to IFN are infected with an isolate of PIV5, termed CPI−, that does not block IFN signalling because it fails to target STAT1 for proteasome-mediated degradation (Chatziandreou *et al.*, 2002a). It is thus possible to infect such ‘IFN-compromised’ cells with CPI− to allow the virus to establish a normal replication pattern and then at various time post-infection (p.i.) to add IFN to the culture medium and monitor the effects of IFN on virus replication and protein synthesis (Carlos *et al.*, 2005). Using this system, we have shown previously that the addition of IFN to cells actively synthesizing CPI− proteins alters the pattern of virus mRNA transcription and induces a marked reduction in virus protein synthesis, particularly of the M and HN proteins (Carlos *et al.*, 2005). Using this system, we showed that myxovirus A (MxA), protein kinase R (PKR) and oligoadenylate synthetase (OAS)/RNase L are not responsible for the alteration in virus protein synthesis observed following IFN treatment (Carlos *et al.*, 2007). Here, we present evidence that IFN-stimulated gene 56/IFN-induced protein with tetratricopeptide repeats 1 (ISG56/IFIT1) is primarily responsible for the observed effects of IFN on PIV5 transcription and protein synthesis.

## Results

### ISG56/IFIT1 is primarily responsible for the IFN-induced alterations to the pattern of PIV5 protein synthesis

**Fig. 1.**
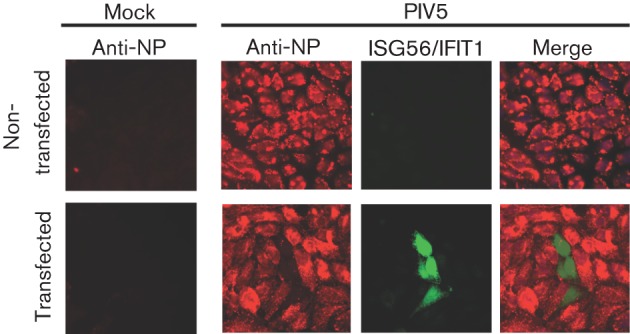
Transient expression of ISG56/IFIT1 inhibits PIV5. Hep2 cells were transiently transfected with a plasmid containing haemagglutinin (HA)-tagged ISG56/IFIT1. At 24 h post-transfection, cells were either mock infected or infected with PIV5 at a high m.o.i. (10 p.f.u. per cell). At 24 h p.i., the cells were fixed and immunostained for the viral NP protein (red) and the HA-tagged ISG56/IFIT1 (green).

We have shown previously that PKR, OAS and Mx are not the major IFN-inducible activities that limit PIV5 replication (Carlos *et al.*, 2007). To attempt to identify the IFN-stimulated gene (ISG) primarily responsible for the IFN-induced inhibition of PIV5, cells were transiently transfected with plasmids permitting the constitutive expression of IFIT3, IFN-induced protein 35 (IFI35), ISG15, ISG20, viperin or ISG56/IFIT1, and the cells were subsequently infected with a variant of PIV5, CPI−, that is unable to block IFN signalling (Chatziandreou *et al.*, 2002a). At 18 h p.i., the cells were fixed and stained for viral NP and for the overexpressed ISGs. Overexpression of IFIT3, IFI35, ISG15, ISG20 and viperin did not have any obvious effect on the levels of NP or P (data not shown). In contrast, and as shown in Fig. 1, cells that transiently expressed ISG56/IFIT1 were negative for NP.

**Fig. 2.**
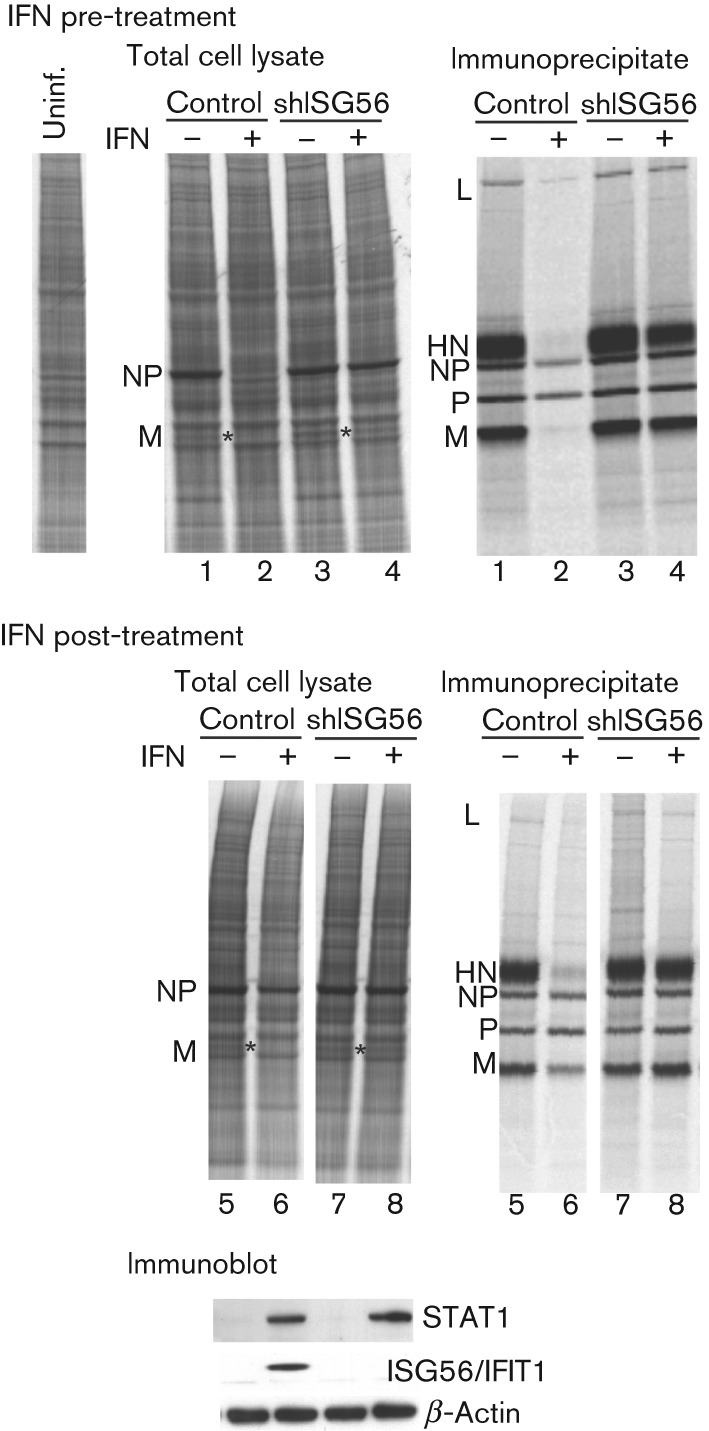
ISG56/IFIT1 is primarily responsible for the IFN-induced changes in CPI− protein synthesis. Hep2/BVDV-N^pro^ (control) cells or Hep2/BVDV-N^pro^.shISG56/IFIT1 (shISG56) cells were treated or not with IFN at 8 h prior to infection with a high m.o.i. of CPI− (IFN pre-treated), or were treated with IFN at 8 h p.i. (IFN post-treatment). At 20 h p.i., the cells were radioactively labelled with L-[^35^S]methionine for 1 h and the viral proteins were immunoprecipitated. Total cell extracts (left-hand panels) and immunoprecipitates (right-hand panels) were separated by electrophoresis through a 4−12 % polyacrylamide gel and the labelled proteins visualized using a phosphoimager. The position of the M protein in the total cell extracts is indicated by asterisks. Samples of the total cell extracts corresponding to the samples in lanes 5–8 were also immunoblotted for the presence of STAT1 and ISG56/IFIT1 (bottom panel), which are inducible by IFN, and for β-actin.

We next knocked down the expression of the endogenous human ISG56/IFIT1 gene in Hep2 and A549 cells using small hairpin RNA (shRNA; Fig. S1, available in JGV Online). The cells used here had been engineered previously to express the bovine viral diarrhea virus (BVDV) N^pro^ protein constitutively, which renders them unable to produce IFN, although they are still able to respond to exogenously added IFN (Carlos *et al.*, 2007; Hilton *et al.*, 2006). When control cells (i.e. those that express BVDV-N^pro^ but not shISG56/IFIT1) were infected with the CPI− strain of PIV5, a normal pattern of virus protein synthesis was observed (Fig. 2, lanes 1). However, virus protein synthesis was dramatically reduced in cells pre-treated with IFN (Fig. 2, lanes 2). In striking contrast, a near-normal pattern of virus protein synthesis was observed in IFN-treated cells with impaired ISG56/IFIT1 expression (Fig. 2, compare lanes 3 and 4).

We also examined the effects of treating cells with IFN subsequent to infection. Again, treatment of control cells with IFN at 8 h p.i. reduced the overall levels of virus protein synthesis, in particular expression of the M and HN proteins (compare lanes 5 and 6 in Fig. 2; the position of the M protein is highlighted by an asterisk in the total cell extracts). When the ISG56/IFIT1-knockdown cells were infected with CPI− and subsequently treated with IFN, a near-normal pattern of virus protein synthesis was again observed (compare lanes 7 and 8 in Fig. 2). Knockdown of ISG56/IFIT1 in naïve Hep2 cells (i.e. without the expression of BVDV-N^pro^), which can produce and respond to IFN and thus begin to inhibit PIV5 prior to the addition of exogenous IFN to the culture medium, also restored a near-normal pattern of virus protein synthesis, even when exogenous IFN was added to the culture medium (Fig. S2). Similar results were also obtained with naïve A549 cells (Fig. S3). These results clearly demonstrated that ISG56/IFIT1 is primarily responsible for the IFN-induced inhibition of virus protein synthesis. It was also striking that the effect of IFN on the levels of virus protein synthesis in Hep2/BVDV-N^pro^ cells was not due to a general shutdown of protein synthesis, as the levels of host protein synthesis were similar in IFN pre-treated, post-treated and untreated cells (Fig. 2; see also Fig. 5).

### IFN, through the activity of ISG56/IFIT1, induces alterations in the pattern of virus transcription

**Fig. 3.**
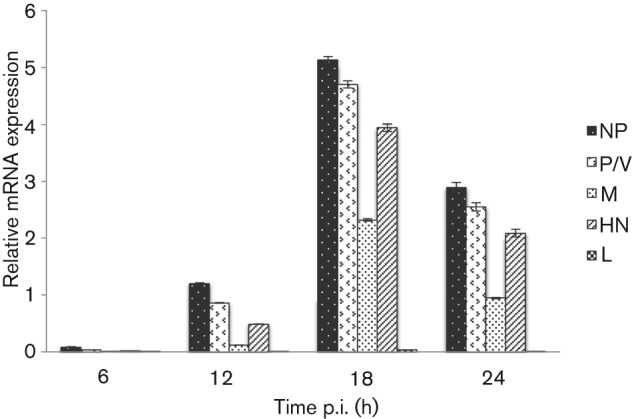
Time course of expression of CPI− mRNA. Hep2/BVDV-N^pro^ cells were infected at a high m.o.i. (10 p.f.u. per cell) of CPI− and at various times p.i., the total cell RNA was extracted and subjected to qPCR for NP, P/V, M, HN and L mRNA. mRNA values are expressed as the quantity of the gene of interest relative to the quantity of β-actin mRNA. Results are shown as means±sem of duplicate samples in two separate experiments.

**Fig. 4.**
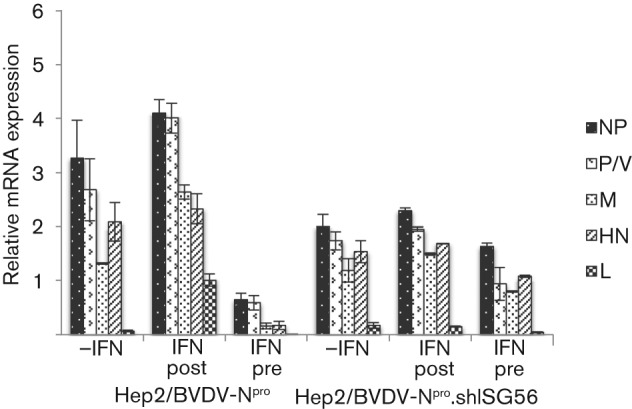
Analysis of the relative levels of CPI− mRNAs in Hep2/BVDV-N^pro^ and Hep2/BVDV-N^pro^/shISG56/IFIT1-infected cells that were pre-treated (IFN pre) or not (−IFN) with IFN at 8 h prior to infection with CPI−, or were treated with IFN at 8 h p.i. (IFN post). At 20 h p.i., total cell RNA was extracted and the levels of NP, P/V, M, HN and L mRNA relative to β-actin mRNA were determined by qPCR. Results are shown as means±sem of duplicate samples in two separate experiments.

We next examined whether the ISG56/IFIT1-induced inhibition of virus protein synthesis was due to inhibition of virus transcription or translation. We first undertook a time-course study of virus mRNA accumulation in the absence of an IFN response in control cells. For this analysis, quantitative PCR (qPCR) was used to measure the relative amounts of NP, P/V, M, HN and L viral mRNAs in CPI−-infected control cells. The results from this analysis (Fig. 3) showed that the highest mRNA levels occurred at around 18 h p.i. and that the general pattern was the same at all time points examined. A clear gradation of mRNA levels for the NP, P, HN and L genes was observed, with L mRNA being the least abundant. As expected, the gradient was related to the order of these genes within the virus genome, consistent with a predictable dissociation of the virus RNA polymerase from the template. The exception to this was M mRNA, which was always significantly less abundant than HN mRNA, despite the more proximal position of the M gene to the 3′ promoter (discussed below). When control cells were treated with IFN at 8 h prior to infection with CPI− and the levels of viral mRNA measured at 20 h p.i., a significant decrease was observed in the amount of all viral mRNAs (Fig. 4). However, surprisingly, treating cells with IFN at 8 h after infection increased, rather than decreased, the amount of all the viral mRNAs, with the greatest increase noted in the amount of L mRNA, suggesting that there was less chance of the polymerase disengaging from the template in cells treated with IFN after infection. Under these conditions, the level of M mRNA was higher than that of HN mRNA (Fig. 4). In contrast to the control cells, treating CPI−-infected Hep2/BVDV-N^pro^/shISG56/IFIT1 cells with IFN either prior to or after infection did not significantly alter either the pattern or levels of virus transcript accumulation.

**Fig. 5.**
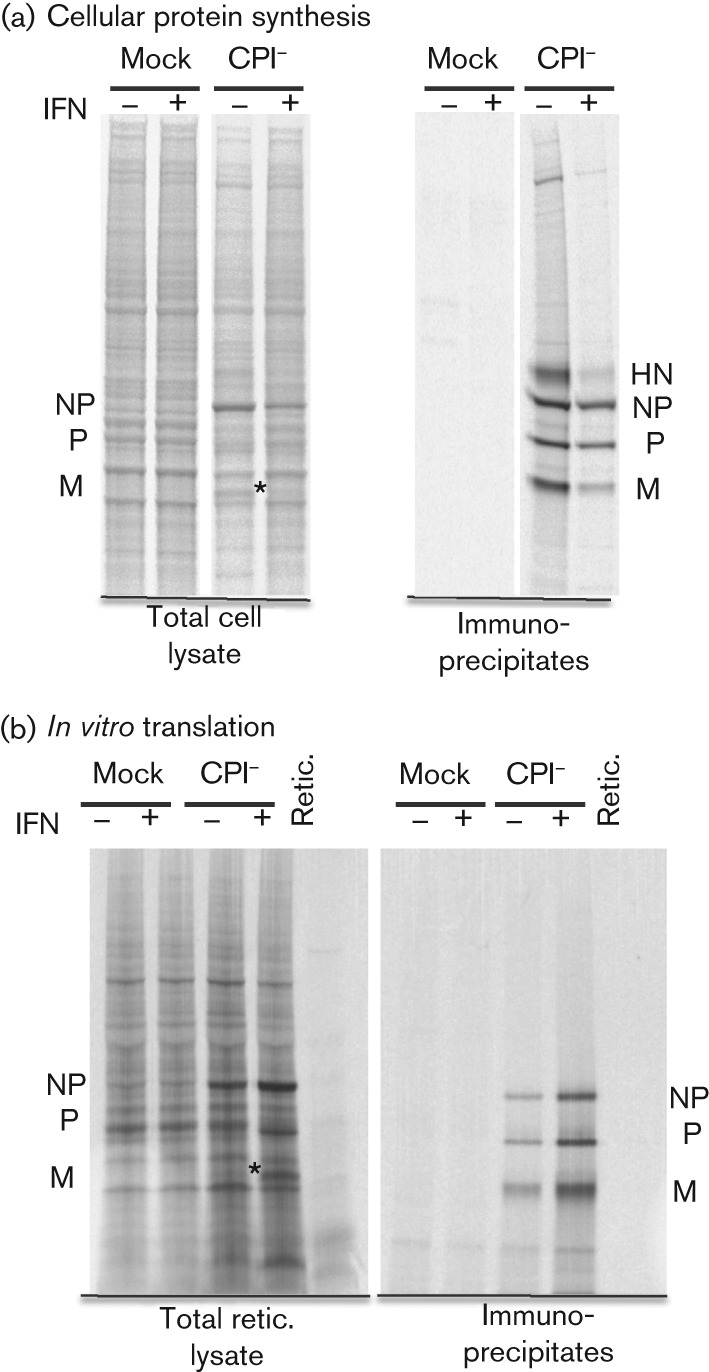
Comparisons of the relative amounts of viral proteins synthesized in infected cells and by *in vitro* translation of mRNA isolated from infected cells. Hep2/BVDV-N^pro^ cells were mock infected or infected with CPI− at a high m.o.i. and treated or not with IFN at 8 h p.i. (a) At 20 h p.i., the cells were radioactively labelled with [^35^S]methionine for 1 h and the viral proteins immunoprecipitated. (b) At 20 h p.i., mRNA was isolated from parallel cultures and subject to *in vitro* translation using a rabbit reticulocyte lysate. Viral proteins were immunoprecipitated or not prior to being separated by electrophoresis through a 7–12 % gradient polyacrylamide gel, and labelled proteins were visualized using a phosphoimager. The position of the M protein in the total cell samples is indicated by asterisks.

The increase in viral mRNA levels observed in control cells treated with IFN after infection was not reflected in the levels of virus protein synthesized, which were significantly reduced in response to IFN (Fig. 2). As, under these conditions, there was no such marked reduction in the synthesis of cellular proteins, these data suggested that IFN, through the action of ISG56/IFIT1, induced a specific block in the translation of viral mRNAs. To investigate this further, mRNA was isolated from CPI−-infected control cells at 20 h p.i. and translated in cell-free rabbit reticulocyte lysate. In order to compare the pattern of virus protein synthesis with that in cells, parallel cultures were also labelled with [^35^S]methionine. In complete contrast to the decrease in virus protein synthesis observed in infected cells, there was an obvious increase in the amount of all the viral proteins made by *in vitro* translation of mRNA isolated from cells that had been treated with IFN (Fig. 5; note the M protein in total cell samples has been highlighted with asterisks). This was consistent with our qPCR data (Fig. 4), which showed that IFN treatment of cells after infection led to an increase in viral mRNA synthesis, and demonstrated that the mRNA remained translatable. In contrast, there was no increase in the *in vitro* translation of cellular proteins, confirming the selective block on viral mRNA translation in CPI−-infected control cells following IFN treatment. Taken together, these results strongly suggested that ISG56/IFIT1 selectively inhibits the translation of viral mRNA.

### PIV5 mRNA and 2′-*O*-methylated caps

**Fig. 6.**
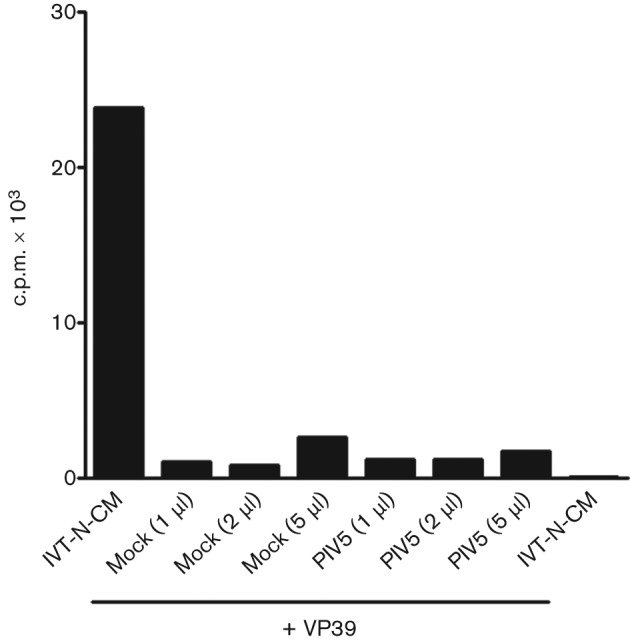
*In vitro* ribose 2′-*O*-methylation assay. Incorporation of ^3^H into poly(A)-containing RNA from mock-infected cells or cells infected with PIV5 after *in vitro* 2′-*O*-methylation with recombinant vaccinia virus 2′-*O*-methyltransferase. *In vitro*-transcribed N7-methylated capped RNA (IVT-N-CM) was used as a positive control for the assay.

It has been suggested recently that the IFIT family proteins ISG56/IFIT1 and ISG54/IFIT2 can recognize and inhibit the translation of (viral) mRNA caps that are not 2′-*O*-methylated and that this can act as a mechanism by which the host cell can distinguish self from non-self mRNA during virus infection (Daffis *et al.*, 2010). Although the PIV5 polymerase is predicted to have 2′-*O*-methyltransferase activity (Ferron *et al.*, 2002), we tested whether the CPI− mRNA cap was methylated at 2′-*O* positions. mRNA was isolated from mock- or CPI−-infected control cells at 20 h p.i. and the incorporation of ^3^H-labelled methyl groups from donor *S*-adenosyl-methionine was monitored by *in vitro* methylation assays using the vaccinia virus 2′-*O*-methyltransferase VP39 (Schnierle *et al.*, 1992). No detectable incorporation of ^3^H was observed in mRNA isolated from mock-infected or CPI−-infected cells (Fig. 6), suggesting that the viral mRNA was indeed 2′-*O*-methylated.

### ISG56/IFIT1 activity indirectly influences the formation of viral cytoplasmic bodies

**Fig. 7.**
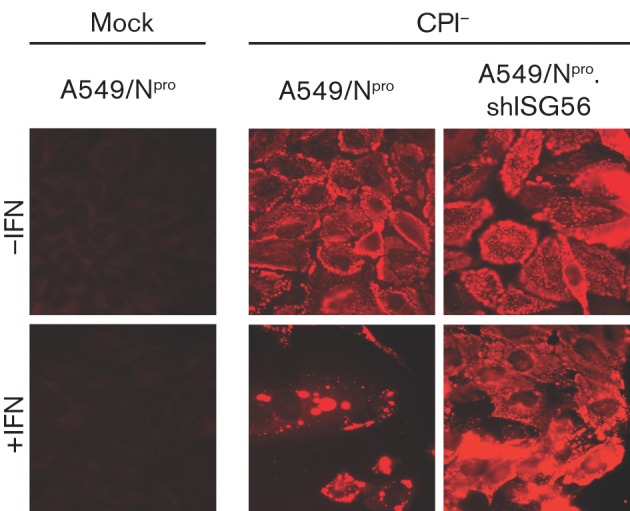
Viral cytoplasmic bodies do not form in response to IFN in cells in which ISG56/IFIT1 has been knocked down. Mock-infected or CPI−-infected A549/BVDV-N^pro^ (A549-N^pro^) and A549/BVDV-N^pro^.shISG56/IFIT1 (A549/N^pro^.shISG56) cells were treated or not with IFN at 8 h p.i. Cells were fixed at 20 h p.i. and the distribution of the viral NP visualized by immunofluorescence.

In addition to inducing alterations in the pattern of virus protein synthesis, IFN also alters the distribution of virus proteins in CPI−-infected cells such that the NP proteins become primarily localized in cytoplasmic bodies (Carlos *et al.*, 2009). We therefore also examined the distribution of the NP and P proteins in naïve and ISG56/IFIT1-knockdown cells. For these experiments, we used A549 cells rather than Hep2 cells because they give better-quality immunofluorescence data. As can be observed in Fig. 7, in untreated control A549 cells (i.e. those that constitutively express BVDV-N^pro^), NP was distributed primarily throughout the cytoplasm, whilst in cells that had been treated with exogenous IFN, NP was located primarily in cytoplasmic bodies. In marked contrast, following treatment of ISG56/IFIT1-knockdown cells with IFN, the distribution of NP was distributed primarily throughout the cytoplasm, resembling the pattern observed in control cells that had not been treated with IFN.

### Although ISG56/IFIT1 is the major ISG that inhibits PIV5, other ISGs also contribute to the antiviral activity of IFN

**Fig. 8.**
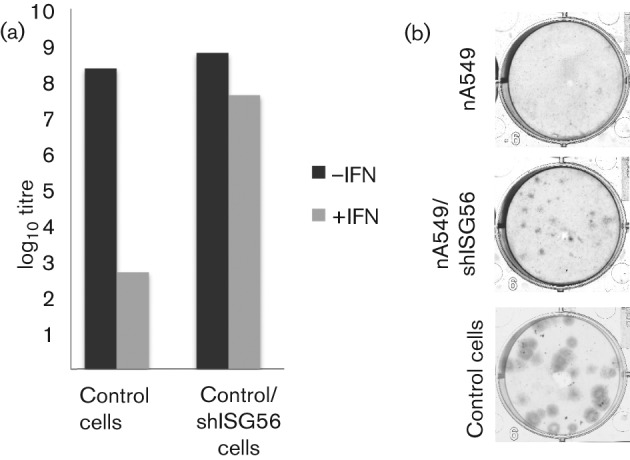
(a) Yield of infectious CPI− virus following a low m.o.i. infection of naïve A549 (control) and A549/shISG56/IFIT1 (control/shISG56) cells. Monolayers of cells in 25 cm^2^ flasks that had been pre-treated or not with IFN at 8 h prior to infection were infected with CPI− at 0.01 p.f.u. per cell and the amount of infectious virus in the culture medium at 48 h p.i. was determined by a plaque assay on Vero cells. (b) Relative plaque size of CPI− on naïve A549 cells (control), naïve A549/shISG56/IFIT1 (nA549/shISG56) and naïve A549/BVDV-N^pro^ cells (nA549). Monolayers of cells in six-well plates were infected with CPI−. At 7 days p.i., the cells were fixed and the virus plaques visualized by immunostaining the monolayers with a pool of mAbs to the NP and P proteins.

To investigate the influence of ISG56/IFIT1 on the production of infectious virus, the amount of infectious virus in the culture medium of CPI−-infected control and ISG56/IFIT1-knockdown cells was determined 2 days after infection (Fig. 8). Pre-treatment of control cells with IFN reduced the titres by ~5 logs (from ~10^8^ to ~10^3^ p.f.u. ml^−1^). In contrast, IFN pre-treatment of the ISG56/IFIT1 knockdown cells only reduced titres by approximately tenfold. These results clearly showed that, whilst ISG56/IFIT1 is a major contributor to IFN-induced inhibition of CPI− growth, other ISGs must also contribute to the induction of an IFN-induced antiviral state. This conclusion was supported by the observation that, whilst knockdown of ISG56/IFIT1 facilitated CPI− plaque formation on naïve A549 cells (which do not normally support CPI− plaques), the plaques that were formed were not as large as those observed on control cells (i.e. those that expressed BVDV-N^pro^; Fig. 8b).

## Discussion

Although PIV5 encodes a powerful IFN antagonist, the V protein, that limits IFN induction and blocks IFN signalling, its ability to circumvent the IFN response is not absolute. Thus, during plaque development, a few virus-infected cells produce IFN, which induces an antiviral state in the uninfected cells surrounding the plaque, thereby slowing the spread of virus (Carlos *et al.*, 2009; Chen *et al.*, 2010). Here, we set out to understand in more detail how IFN induces an antiviral state to PIV5 by identifying the ISGs that inhibit virus replication. The results presented here demonstrated that, although other ISGs contribute to a minor degree to the IFN-induced antiviral state, remarkably ISG56/IFIT1 is primarily responsible for the observed IFN-induced alterations in PIV5 transcription and protein synthesis.

ISG56/IFIT1 is a member of a family of proteins whose expression is strongly induced from very low basal levels by IFN, and by activated IRF-3 (reviewed by Fensterl & Sen, 2011). IFIT1 family members have structural features in common: all contain several tetratricopeptide repeats representing helix–turn–helix motifs that mediate a variety of protein–protein interactions. The best-studied cellular function of ISG56/IFIT1 is the inhibition of translation initiation (Guo *et al.*, 2000; Terenzi *et al.*, 2005) that is mediated through its binding to eukaryotic initiation factor 3 (eIF3). In addition to a general inhibition of mRNA translation, it has been suggested recently that ISG56/IFIT1 specifically inhibits the translation of viral mRNAs that are not appropriately capped. Eukaryotic host mRNAs are modified at the 5′ end by the addition of a 5′–5′-linked, non-templated guanosine residue, which is methylated at N7, and additionally have 2′-*O*-methylation of the ribose ring of the first one or two templated bases. Whilst N7-methylation of the guanosine increases translational efficiency, the reason for 2′-*O*-methylation of the ribose sugars is unclear (Furuichi & Shatkin, 2000). However, recent work has suggested that lack of methylation of the 2′ ribose might allow cells to distinguish self from non-self RNA (Daffis *et al.*, 2010; Pichlmair *et al.*, 2011; Züst *et al.*, 2011). To avoid this recognition, it is suggested that viruses that replicate in the cytoplasm have evolved both N7- and 2′-*O*-methyltransferases to methylate their viral mRNA cap structures. West Nile virus, mouse coronavirus and vaccinia virus mutants lacking 2′-*O*-methyltransferase activity have enhanced sensitivity to IFN and the antiviral activity of IFIT1 proteins (Züst *et al.*, 2011). It has also been reported recently that ISG56/IFIT1 binds uncapped triphosphorylated RNA, which is found at the 5′ ends of the genomic and antigenomic RNA of some RNA viruses, as well as at the ends of some viral transcripts (Pichlmair *et al.*, 2011). However, the relationship between this property and the ability to preferentially inhibit translation of mRNA with cap structures lacking 2′-*O*-methyl groups but still having an N7-methylguanosine (and which therefore cannot be triphosphorylated) is unclear.

The results presented here showed that ISG56/IFIT1 selectively inhibited PIV5 mRNA translation. Our inability to detect any un-2′-*O*-methylated mRNA within the sensitivity of the test suggested that the selective inhibition is unlikely to be due to any deficiency in cap 2′-*O*-methylation, although we cannot rule out the possibility that cells may contain small amounts of CPI− mRNAs that are not 2′-*O*-methylated, which may activate ISG56/IFIT1. In this regard, it is of note that, whilst the PIV5 viral polymerase has the absolutely conserved residues for 2′-*O*-methyltransferase enzymes (Ferron *et al.*, 2002), PIV5 (like Newcastle disease virus, parainfluenza virus type 2, mumps virus and simian virus 41) has an alanine instead of the first glycine (position G1804 in Sendai virus L) in a glycine-rich motif in the methyltransferase domain of other members of the order *Mononegavirales* that may influence the efficiency of 2′-*O*-methylation (Murphy *et al.*, 2010).

As well as selectively inhibiting translation of PIV5 mRNAs, it is also clear that ISG56/IFIT1 affects virus transcription. In support of the data presented here, using Northern blot analysis to examine the effects of IFN on the transcription of CPI− mRNAs in Vero cells (which cannot produce but can respond to IFN), we reported that there was an increase in NP and P mRNA transcription following IFN treatment p.i. (Carlos *et al.*, 2005). How ISG56/IFIT1 activity affects virus transcription remains to be established. It may be that the ISG56/IFIT1-mediated inhibition of viral protein synthesis leads indirectly to an alteration in the pattern of virus transcription. For example, a change in the ratio of a particular virus protein to other viral proteins [e.g. the V protein, which has a relatively short half life (Fearns *et al.*, 1994) and negatively regulates viral RNA synthesis (Lin *et al.*, 2005)] results in an increase in virus transcription and/or alterations to the processivity of the viral polymerase–transcription complex. Alternatively, it may be that ISG56/IFIT1 directly interacts with the viral polymerase, altering its activity. In this regard, it is of note that ISG56/IFIT1 binds to and inactivates the viral E1 helicase of human papillomavirus, which is essential for viral DNA replication (Terenzi *et al.*, 2008).

In our previous studies, which used Northern blot analysis to examine the pattern of CPI− transcription in Vero cells treated with IFN p.i., as well as noting an increase in the levels of NP and P mRNA, we also noted a significant decrease in the levels of full-length HN mRNAs (Carlos *et al.*, 2005). At the time, we speculated that this was either because the polymerase was more likely to disengage from the template as it proceeded down the genome in cells treated with IFN, or that IFN somehow affected the stability of the HN mRNA. Given that we have shown that, in IFN-treated PIV5-infected control Hep2 cells, there is an increase in the levels of all the viral mRNAs, this suggests that the latter explanation may have been correct. Alternatively, there may be a difference in the effects of IFN on Vero and Hep2 cells.

We also suggested previously that IFN may modulate and influence the establishment and maintenance of persistent infections with PIV5 by favouring the formation of virus cytoplasmic bodies (which have been shown to contain genomic RNA), and that these may be sites at which the virus can establish a quiescent infection, hiding both from innate intracellular antiviral responses and adaptive immune responses (Carlos *et al.*, 2009; Chatziandreou *et al.*, 2002b; Fearns *et al.*, 1994). In this respect, it is of interest that the most marked reduction in the viral proteins synthesized in cells either pre- or post-treated with IFN encoded proteins downstream of the P gene, in particular the M protein, whilst the synthesis of NP and P, although inhibited, remain relatively high. The reason for these apparent differences in the effects of IFN on the relative inhibition of the different viral proteins remains unclear but may simply reflect the stability of the proteins, with NP and P being very stable and M and HN relatively unstable. Nevertheless, a consequence of this change in the relative levels of the viral proteins (i.e. the increase in the relative amounts of NP and P compared with the other virus proteins) might favour the formation of cytoplasmic bodies. Indeed, the fact that knockdown of ISG56/IFIT1 prevented the formation of inclusion bodies, and that inducible co-expression of NP and P in cell lines leads to the formation of large cytoplasmic aggregates (in the absence of an IFN response; Precious *et al.*, 1995), suggests that it is the balance of viral proteins that are synthesized that influences the formation of viral cytoplasmic bodies rather than the induction of a specific cellular ISG that drives their formation.

## Methods

### 

#### Cells, viruses and IFN.

Vero, Huh7, A549 and Hep2 cells and their respective derivatives were grown as monolayers in 25 or 75 cm^2^ tissue-culture flasks in Dulbecco’s modified Eagle’s medium supplemented with 10 % fetal calf serum at 37 °C. When needed, cells were treated with human recombinant IFN (Roferon-A; Roche) at 1000 U ml^−1^. The CPI− isolate was grown and titrated in Vero cells.

#### Preparation of radiolabelled antigen extracts, immunoprecipitation and SDS-PAGE.

The methodology used has been described elsewhere (Carlos *et al.*, 2005). Briefly, cells were mock infected or infected with CPI− and at 8 h p.i. were treated with IFN or left untreated for 12 h and then metabolically labelled for 1 h with l-[^35^S]methionine (500 Ci mmol^−1^; MP Biomedical). After labelling, the cells were lysed in immunoprecipitation buffer, sonicated and centrifuged for 30 min at 12 000 ***g*** to remove solid material. Immunocomplexes were formed by incubating soluble antigens with protein G–Sepharose (Sigma), pre-coupled with a mix of mAbs to the viral proteins NP, P, HN and M. The proteins in immunocomplexes were dissociated by heating for 5 min at 100 °C and analysed by SDS-PAGE. The gels were fixed, stained and dried, and the resolved bands visualized by phosphorimage analysis.

#### Plasmids and generation of cells expressing shRNA to ISG56/IFIT1.

The mammalian expression vector containing the full-length haemagglutinin (HA)-tagged IFIT1/ISG56 gene was a kind gift from Dr F. Weber (Institute for Virology, Philipps University Marburg, Germany). Lentivirus vector used for the expression of ISG56/IFIT1 shRNA sequence was based on pLKO.1puro, as described by Everett *et al.* (2007). A derivative of the vector expressing the blasticidin resistance gene (pLKO.BLAST) was obtained from Dr R. D. Everett (CVR, University of Glasgow, UK). Double-stranded oligonucleotides corresponding to the target sequences were cloned into pLKO.BLAST. The target sequences for human ISG56 were for region 1 (5′-GGATAAAGCTCTTGAGTTA-3′) and region 2 (5′-CTACAAATTGGAAGGAAAT-3′) (Li *et al.*, 2009). The method used to isolate lentivirus stock has been described previously (Hilton *et al.*, 2006). Lentivirus-infected cells were selected with blasticidin (10 µg ml^−1^) and maintained for 4 weeks in medium containing blasticidin.

#### Immunofluorescence.

For immunofluorescence analysis, cells were grown on 10 mm diameter coverslips (General Scientific Co.) in individual wells of six-well plates. Cells were transiently transfected with a plasmid encoding HA-tagged ISG56/IFIT1. At 24 h post-transfection, cells were infected with the different viruses. At 24 h p.i., monolayers of cells on coverslips were incubated in fixing solution (5 % formaldehyde in PBS) for 15 min at room temperature, permeabilized (0.5 % NP-40, 10 % sucrose in PBS) for 10 min and washed three times in PBS containing 1 % FCS. To detect the proteins of interest, cell monolayers were incubated with 20 µl of appropriately diluted primary antibody for 1 h. For CPI− infection, mAbs to NP and P proteins were used, named SV5-NP-a and SV5-P-e, respectively (Randall *et al.*, 1987). To detect overexpressed ISG56/IFIT1, anti-HA rabbit polyclonal antibody (Sigma) was used. After subsequent incubation with primary antibody, the cells were washed several times with 1 % FCS in PBS and incubated for 1 h with secondary Texas Red-conjugated goat anti-mouse IgG (for viral proteins) or FITC-conjugated goat anti-rabbit IgG for ISG56/IFIT1 detection (Sera Lab).

#### qPCR.

Total cellular RNA was extracted from infected cells using TRIzol (Invitrogen). Two micrograms of RNA was subsequently used for RT-PCR. Fragments of ~200 bp were amplified with primers for NP, P, M, HN and L. β-Actin was used as a reference gene. Primer sequences were: NP: 5′-AGGGTAGAGATCGATGGCT-3′ (forward) and 5′-GTCTGACCACCATTCCCTT-3′ (reverse); P: 5′-AATACCACCAGGGGTCACAG-3′ (forward) and 5′-CGAGCACCCAAACTGTGCTT-3′ (reverse); M: 5′-TCATGAGCCACTGGTGACAT-3′ (forward) and 5′-TGGAATTCCCTCAGTTGTCC-3′ (reverse); HN: 5′-AACTCTGCAGTCGCTCTACC-3′ (forward) and 5′-GCAATCTGACACTTGGCCCA-3′ (reverse); L: 5′-TCCAAGTGATGACTTTGAATT-3′ (forward) and 5′-CCATACTCATTACTCGTGTGCC-3′ (reverse); and β-actin: 5′-ACCAACTGGGACGACATGGAG-3′ (forward) and 5′-TAGCACAGCCTGGATAGCAAC-3′ (reverse). The plotted values of mRNA were expressed as the quantity of the gene of interest relative to the quantity of the reference gene, to obtain normalized and relative expression values. Each sample was performed in duplicate on the same qPCR plate in two separate experiments. A non-template sample and a non-reverse transcriptase sample were analysed routinely as negative controls. Data were collected using a 7300 Real-time PCR System (Applied Biosystems).

#### *In vitro* methylation assay.

Poly(A)-containing RNA was isolated from 1.5×10^7^ mock- or CPI−-infected A549 cells with an Oligotex Direct mRNA Mini kit according to the manufacturer’s instructions (Qiagen). *In vitro*-transcribed N7-methyl-capped model RNA (m7-Cap-RNA) encoding the NP of mouse hepatitis coronavirus was generated using a T7 RiboMax Express Large Scale RNA Production System (Promega). The N7-methylated cap was added using the ScriptCap m7G Capping System according to the manufacturer’s recommendation (Cellscript). 2′-*O*-Methylation reactions were carried out as described previously (Züst *et al.*, 2011). Briefly, 100 ng of *in vitro*-transcribed m7-Cap-RNA or 300–1500 ng poly(A)-containing RNA derived from virus-infected cells or the corresponding amount of poly(A)-containing RNA from uninfected cells was incubated for 1 h at 37 °C with ScriptCap 2′-*O*-methyltransferase (Cellscript) in the presence of 0.5 µM *S*-adenosylmethionine and 1.4 µM ^3^H-labelled *S*-adenosylmethionine (78 Ci mmol^−1^; Perkin Elmer). Reactions were purified with SigmaSpin Post-Reaction Clean-Up columns (Sigma-Aldrich) and eluates were mixed with 2 ml Ultima Gold scintillation fluid for measurement of ^3^H incorporation with a Packard Tri-Carb liquid scintillation counter (Perkin Elmer).
